# Spatio-Temporal Distribution of Environmental Health Risk of Heavy Metals in Industrial Wastewater of China during 1999–2018

**DOI:** 10.3390/ijerph18115920

**Published:** 2021-05-31

**Authors:** Ruru Han, Beihai Zhou, Huilun Chen

**Affiliations:** School of Energy and Environmental Engineering, University of Science and Technology Beijing, Beijing 100083, China; hanruru@126.com (R.H.); zhoubeihai@sina.com (B.Z.)

**Keywords:** heavy metals, industrial wastewater, environmental health risk, spatio-temporal distribution

## Abstract

In recent decades, environmental health risk caused by heavy metals in industrial wastewater (EHR-IHM) has become a serious issue globally, especially for China. Given the spatial difference of heavy metal emissions, hydrogeography, population distribution, etc., it is essential to estimate China’s EHR-IHM from a high-resolution perspective. Based on the framework of USEtox, this study constructs an environmental health risk assessment method for heavy metals discharged from industrial wastewater by coupling the Pollutant Accumulation Model (PAM). This method also considers the process of heavy metal flows between upstream and downstream areas. Based on this constructed method, we investigate the spatio-temporal distribution of EHR-IHM of As, Cd, Cr(VI), Hg, and Pb in China from 1999 to 2018. Results showed that the EHR-IHM in China increased rapidly during 1999–2007 and decreased gradually during 2007–2018, with the highest Damage Level (DL) of 6.8 × 10^4^ disability-adjusted life years (DALY). As and Cr(VI) were the major heavy metal pollutants, which induced 58.9–70.6% and 23.9–36.2% of the total EHR-IHM, respectively. Intake of aquatic products was the dominant exposure route, accounting for over 84.1% of national EHR-IHM, followed by drinking water intake, accounting for 9.5–15.8%. Regarding spatial distribution, the regions with high EHR-IHM are mainly distributed in the middle–lower reaches of the Yangtze River, southeast coastal cities, Bohai Rim, etc.

## 1. Introduction

Environmental health risk caused by heavy metals discharged from industrial wastewater (EHR-IHM) has become a worldwide concern since the Minamata and Itai-Itai diseases in Japan [1]. In recent years, the EHR-IHM has become increasingly prominent in China, along with the intensified industrial production activities. More than 60 heavy metal pollution incidents occurred in China during 2004–2013, among which over 90% were caused by heavy metal accumulations [2]. It has been discovered that the heavy metal accumulation effects are severe in rivers of China, such as the Yangtze River [3], the Pearl River [4,5], and the eastern coastal rivers [6]. Although heavy metal emissions have decreased significantly in the recent decade, EHR-IHM is still prominent due to a large amount of historically accumulated heavy metal [7,8]. Since the 13th Five-Year Plan period (2015–2020), preventing environmental risks and protecting public health have become the focus of comprehensive prevention and control of heavy metal pollution in China. Assessing EHR-IHM considering historically accumulated industrial heavy metals is vital in achieving this goal.

Previous studies mainly focused on a local scale with investigation of heavy metal pollution and risk in China [9]. However, for China’s goal of prevention and control of health risks caused by heavy metals, there lacks EHR-IHM evaluation on the national scale. Besides, there are apparent spatial heterogeneities considering heavy metal emissions, hydrogeography, social environment, etc., in China, making high-resolution spatial investigation critical. The effects of spatial heterogeneities have been uncovered in foreign countries. For example, for heavy metal pollution, Somma et al. studied the long-lasting arsenic pollution over sediment geochemistry in polluted areas [10], and Buccino et al. studied the impact of historical industrial activities on arsenic marine sediments pollution [11]. As for health risks, Manneh et al. show that the spatial variability of toxic organic chemicals’ intake fractions among Canadian sub-watersheds (172 zones) was up to 10 orders of magnitude, implying that higher spatial resolution could promote the reliability of the assessment results [12]. Besides, a variation of 3 orders of magnitude for human intake was found in some chemical assessments in Western Europe [13]. More importantly, these two studies also show that the watershed unit is suitable for investigating the spatial variability on the environmental migration and human ingestion exposure of chemicals. The watershed is a natural region with solid integrity, and the upstream and downstream areas are closely interrelated [14]. Inevitably, the emission and pollution of heavy metals in upstream areas would directly affect the downstream area [15], making it a critical factor influencing human health risks. However, existing EHR-IHM assessments have not considered the interrelations between upstream and downstream regions.

General EHR-IHM assessment models of environmental health risk caused by heavy metals consider migration among environmental media, human exposure, and damage to public health. The most popular models and tools include CML2001 [16], Eco-Indicator 99 [17], EDIP 2003 [18], IMPACT2002+ [19], Stepwise 2006 [20], ReCiPe [21], USEtox [22], TRACI [23], etc. Among these models, USEtox is the harmonized meeting point of different existing previous models. Due to the consensus behind it, it provides a high level of transparency, consistency, and reliability, which provides a relatively complete analytical framework and parameters. USEtox is widely accepted for assessing human health risks caused by heavy metal emissions [24], and is recommended by the United Nations Environment Program Society for Environmental Toxicology and Chemistry (UNEP/SETAC) for assessing the toxic effects of heavy metals. However, USEtox is developed for national and continental assessment and only has one unified set of parameters for China. Thus, it is difficult for USEtox to reflect the spatial heterogeneity of EHR-IHM in China [25].

To fill these knowledge gaps, this study develops the multimedia migration model of heavy metals in industrial wastewater (MM-IHM) based on the framework of USEtox, coupling the Pollutant Accumulation Model (PAM). To support practical management, Cadmium (Cd), hexavalent chromium (Cr(VI)), mercury (Hg), lead (Pb), and arsenic (As), listed as priority-controlled heavy metal (metalloid) pollutants in the 12th Five-Year Plan for Comprehensive Prevention and Control of Heavy Metal Pollution in China [26], are considered in this model. In particular, as a metalloid, As is also included in this campaign and referred to as a metal for simplicity of writing. More importantly, we take the process of heavy metal stream flows from upstream to downstream areas into consideration. With a high-resolution parameter dataset for MM-IHM reflecting China’s geographical and environmental characteristics, we estimate the exposure and induced EHR-IHM in each year during 1999–2018 at the scale of tertiary watersheds, which is the third-level subdivisions of watersheds in China. The uncovered spatio-temporal distribution could support the systematic and refined management of HM-EHR in China.

## 2. Methods and Data

### 2.1. The Framework of the EHR-IHM Assessment Method

**Figure 1:**
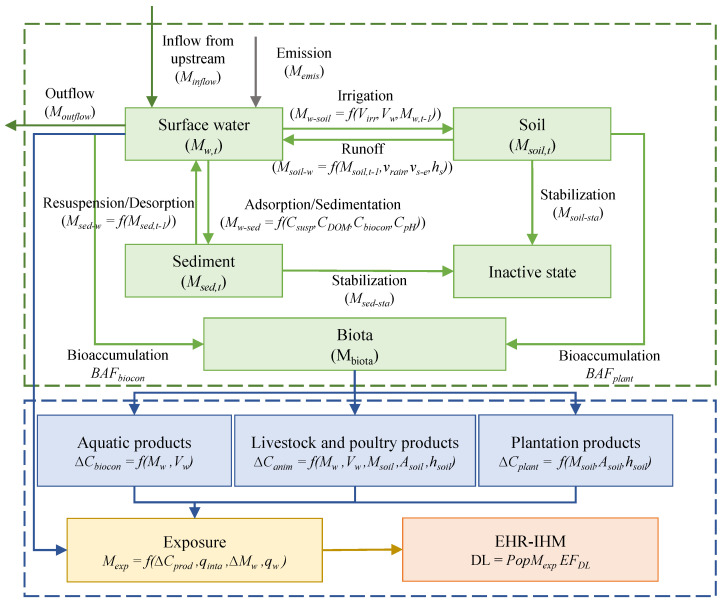
The framework of the EHR-IHM assessment method.

When released into the water body, heavy metals keep a dynamic equilibrium in three forms (dissolved, granular, and sedimentary [27]), migrate among environmental media of water, sediment, and soil, or outflow to the downstream areas. Then, heavy metals are transferred to aquatic organisms, plants, and animals through bioaccumulation. Finally, human exposure to heavy metals causes damage to public health through the intake of these agricultural products, which can be characterized by disability-adjusted life years (DALY). The framework is displayed in Figure 1.

### 2.2. The Multimedia Migration Model of Heavy Metals in Industrial Wastewater (MM-IHM)

This study couples the PAM with USEtox to uncover the heavy metal transitions in environmental media. The PAM was developed to simulate long-term changes of heavy metal concentrations in environmental media based on the mass balance theory [28]. Based on this model, the accumulation of heavy metals in surface water, agricultural soils, and sediment caused by industrial wastewater heavy metal emissions can be evaluated through analyzing various input and output pathways. As displayed in Figure 1, the migration process of discharged heavy metals related to the media of surface water includes ① dissolved metals absorbed to suspended particulate matter and then settled down or adsorbed to the sediment, ② re-suspended or absorbed to dissolved metals from sediment, ③ transferred to soil by irrigation with water containing dissolved and suspended heavy metals, ④ return to surface water from the soil through rainfall erosion, ⑤ bioaccumulation, and ⑥ outflows to downstream areas.

Therefore, inputs of heavy metals to surface water include ① local emissions, ② inflows from upstream, ③ re-suspended or absorbed to dissolved metals from sediment after settled down or adsorbed, as well as ④ return to surface water from the soil through rainfall erosion after being transferred to agricultural soil. Meanwhile, output pathways of heavy metals include: ① settled down or adsorbed to the sediment, ② transferred to agricultural soil by irrigation, ③ bioaccumulation by the aquatic organism and then removed from the system, and ④ outflows to downstream areas. Therefore, the mass of heavy metals in surface water (*M_w_*) can be calculated as Equation (1):

(1) $$\begin{array}{ll} M_{w,t}=M_{w,t-1}+ & M_{\mathrm{inputs}}+M_{\mathrm{outputs}}\\ & =M_{w,t-1}+M_{emis,t}+M_{inflow,t}+M_{sed-w,t}+M_{soil-w,t}-M_{w-soil,t}\\ & -M_{w-sed,t}-M_{biocon,t}-M_{outflow,t} \end{array}$$

where the *M_w,t_* and *M*_*w,t*−1_ represent the mass of heavy metals in the time of *t* and *t* − 1, respectively (kg); *M_emis,t_*, *M_inflow,t_*, *M_outflow,t_*, and *M_biocon,t_* represent the mass of emissions, inflows from upstream, outflows to downstream, bioaccumulation of heavy metals, respectively (kg); *M_w-sed,t_* represents the mass of heavy metals settled down and adsorbed to the sediment (kg); *M_sed-w,t_* represents the mass of heavy metals re-suspended or desorbed to dissolved metals from sediment (kg); *M_w-soil,t_* represents the mass of heavy metals transferred to soil by irrigation (kg), and *M_soil-w,t_* represents the abundance of heavy metals return to surface water from soil.

The outflows of heavy metals in the upstream watershed were the inflows of its downstream watershed, evaluated with Equation (2):

(2) $$M_{outflow,t}=\left(M_{w,t-1}+M_{emis,t}+M_{inflow,t}-M_{w-sed,t}+M_{sed-w,t}-M_{w-soil,t}\;+M_{soil-w,t}-M_{biocon,t}\right)\frac{V_{outflow,t}}{V_{w,t}}$$

where *V_outflow,t_* represents the volume of runoff outflow (m^3^), and *V_w,t_* represents the gross amount of water resources (m^3^).

In addition to *M_w-soil,t,_* and *M_soil-w,t_*, the mass of heavy metals in the soil also depends on the abundance of heavy metals in the time of *t* − 1 (*M*_*soil,t*−1_), when heavy metals have been removed by harvesting plants absorbing heavy metals (*M_plant,t_*) and infiltrated into the stable layer due to leaching (*M_soil-sta,t_*), as displayed in Equation (3):

(3) $$M_{soil,t}=M_{soil,t-1}+M_{w-soil,t}-M_{soil-w,t}-M_{plant,t}-M_{soil-sta,t}$$

As a secondary source, the mass of heavy metals in sediments can be calculated with *M_w-sed,t_*, *M_sed-w,t_*, and the abundance of heavy metals that sink into the inactive state (*M_sed-sta,t_*), as displayed in Equation (4):

(4) $$M_{sed,t}=M_{sed,t-1}+M_{w-sed,t}-M_{sed-w,t}-M_{sed-sta,t}$$

Meanwhile, *M_w-sed,t_*, *M_sed-w,t_*, *M_w-soil,t_*, *M_biocon,t_*, and *M_soil-w,t_* are calculated with the method listed in the USEtox manual with parameters in the scale of the tertiary watershed. The *M_w-sed,t_* is related to the concentration of suspended particulate matter, dissolved organic matter (DOM) and biota in surface water, and pH value. Due to insufficient data of DOM and biota, and minor variation of pH nationwide, default values for China in USEtox are used. The *M_w-soil_* is related to the irrigation volume with surface water and the heavy metal concentration, and *M_soil-w_* is associated with the hydraulic erosion of soils and heavy metal concentration.

### 2.3. Exposure and Risk Assessment Model of EHR-IHM

This study uses the indicator of Damage Level (*DL*) to characterize EHR-IHM, which is calculated with factors of exposure dose (*M_exp_*), duration of exposure (*n*), the population density (Pop), and toxicity effect of heavy metals (*EF*), as displayed in Equation (5):

(5) $$DL=Pop\times n\underset{x}{\sum }\underset{i}{\sum }EF_{x,i}\times M_{exp,x,i}$$

where *DL* represents the Damage Level caused by heavy metal emissions from industrial wastewater (DALY), *EF_x,i_* means the effect factor of heavy metal *i* through exposure route *x* (DALY/g), *M_exp,i_* indicates the intake dose of heavy metal *i* through exposure route *x* (g/person/day), *n* represents the duration of exposure (day), and *Pop* represents the population (person).

Dietary intake is the main route of heavy metal exposure, while the dermal pathway is considered negligible [29]. The exposure dose depends on the intakes of all kinds of agricultural products and heavy metal contents, as Equation (6) shows:

(6) $$M_{exp,i}=\sum ∆C_{inta,i,k}q_{inta,i,k}$$

where *C_inta,i,k_* represents the content of heavy metal *i* in agricultural product *k*, and *q_inta_* represents the intakes of agricultural product *k*. According to the dietary pattern in China, the dominant agricultural foods include rice, wheat, vegetables, pork, poultry, milk, and aquatic products [30]. These foods can be categorized into plant products, aquatic products, as well as livestock and poultry products, according to cultivation pattern (Table 1).

Heavy metals in plant products are mainly accumulated from agricultural soils, while aquatic products accumulate heavy metals from surface water. Bioaccumulation of heavy metals in livestock and poultry products is related to heavy metal contents in soil, water, and plants. Heavy metal contents in these foods can be estimated with Equations (7)–(9):

(7) $$∆C_{biocon}=BAF_{biocon}∆C_{w}$$

(8) $$∆C_{plant}=BAF_{plant}∆C_{soil}$$

(9) $$∆C_{anim}=\sum BAF_{anim}C_{x}=BAF_{w-anim}∆C_{w}+BAF_{soil-anim}∆C_{soil}\;+BAF_{plant-anim}∆C_{plant}$$

where ∆*C_biocon_*, ∆*C_anim_*, and ∆*C_plant_* represent the mean heavy metal contents in aquatic products, livestock and poultry products, and plant products, respectively (g/kg); *BAF_biocon_* and *BAF_plant_* represent the bioaccumulation factor of aquatic products and plant products, respectively; *BAF_w-anim_*, *BAF_soil-anim_*, and *BAF_plant-anim_* represent the bioaccumulation factors of livestock and poultry products from water, soil, and plant products, respectively.

The mean distribution contents of heavy metals (∆*C_soil,t_*) in soil and surface water (∆*C_w,t_*) can be estimated with Equations (10) and (11), respectively:

(10) $$∆C_{soil,t}=\frac{M_{soil}}{A_{soil},h_{soil}}$$

(11) $$∆C_{w,t}=\frac{M_{w,t}}{V_{w,t}}$$

where *A_soil,t_* represents the area of agricultural soil (km^2^), and *h_soil_* represents the depth of agricultural soil.

### 2.4. Data

The most important parameters used to estimate the spatial variability and related data sources are summarized in Table 2. The rest of the parameters are set according to USEtox. We used ArcGIS 10.3 to manage, process, and visualize data and the results.

Huang et al. have constructed a high-resolution grid dataset (at 1 × 1 km scale) of heavy metals emissions (As, Cd, Cr(VI), Hg, and Pb) from industrial wastewater in China during 1998–2015 [31,36]. The dataset of heavy metal emissions from industrial wastewater in 1999–2018 was obtained through missing data interpolation based on this dataset construction method. In the same way, the streamflow dataset was brought about based on Lin et al. [33]. The dietary exposure data in Highlights of the Chinese exposure factors handbook (Children) and Highlights of the Chinese exposure factors handbook (Adult) was the average data of provincial administrative units [34,35], and the dietary intake data of each tertiary watershed are shown in Table S1 in Supplementary Materials. The dataset in the tertiary watershed-scale was constructed based on spatially weighted calculations with ArcGIS.

## 3. Results and Discussion

### 3.1. Temporal Trend of EHR-IHM

**Figure 2:**
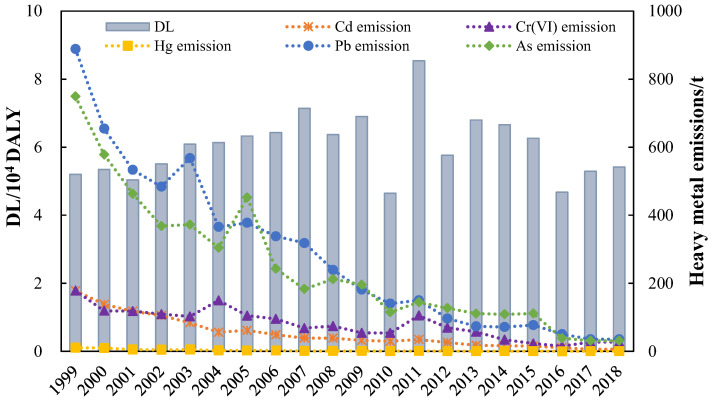
The DL of heavy metal emissions from industrial wastewater in 1999–2018.

During 1999–2018, EHR-IHM in China increased during 1999–2007 and peaked at 8.5 × 10^4^ DALY in 2011 (Figure 2). The highest DL in 2011 was attributed mainly to the rise in heavy metal emissions. Compared with those in 2010, the emissions of Cd, Cr(VI), Hg, Pb, and As in industrial wastewater increased by 16.9%, 93.9%, 15.9%, 7.1%, and 25.3% in 2011, respectively. Although the heavy metal emissions from industrial wastewater decreased significantly, the DL increased from 5.2 × 10^4^ DALY in 1999 to 7.1 × 10^4^ DALY in 2007 due to the accumulative effect of heavy metals. The EHR-IHM in 2010 was the lowest, and the DL was only 83.7% of the value in 2009. This was mainly induced by a significant decrease in heavy metal emissions from industrial wastewater. In particular, the emissions of As and Pb in industrial wastewater reduced by 41.1% and 22.6%, respectively. Besides, 2010 was a rainy year, when the amount of surface water resources was 28.9% more than that of 2009. As a result, the concentration of heavy metals in various environmental media and ingested products was low, so people were exposed less than in other years [37]. The EHR-IHM fluctuated after 2012. The DL was 5.4 × 10^4^ DALY in 2018, which was 63.5% of that in 2011.

### 3.2. Primary Heavy Metals

**Figure 3:**
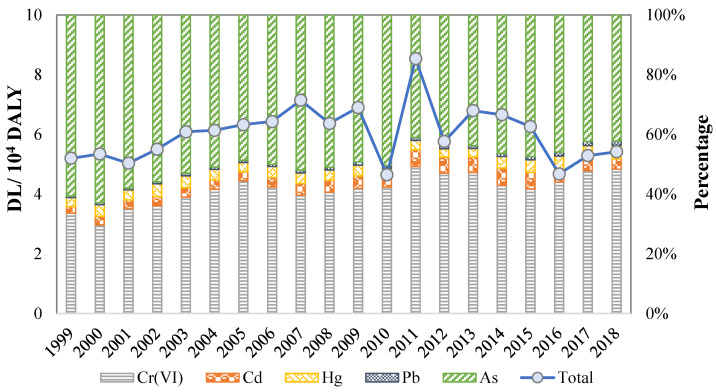
The contribution proportions of DL of five heavy metals.

As displayed in Figure 3, As and Cr(VI) contributed most to the total DL, with the proportions of 42.1–62.9% and 29.7–48.4%, respectively. From 1999 to 2018, the proportion of Cr(VI) in total DL increased gradually, while As decreased obviously. Meanwhile, the ratio of DL caused by Cd emission from industrial wastewater increased and reached the highest value (5.9%) in 2014, and then gradually decreased to 3.5% in 2018. The contribution of Hg to DL fluctuated in 1999–2018, with the highest value of 4.8% in 2017. The contribution proportion of Pb was 0.6–1.5% and showed a slight upward trend in 1999–2018. Consistent with existing studies, As and Cr(VI) are the most prominent heavy metal pollutants due to their high toxicity. For example, As pollution in the surface water of Xiangjiang River was the most serious, which contributed the most to both the non-carcinogenic risk and carcinogenic risk [38], and industrial wastewater discharge is one of the primary sources of As, Cd, Pb, and Hg in the surface water in this area.

### 3.3. Dominant Exposure Pathways

**Figure 4:**
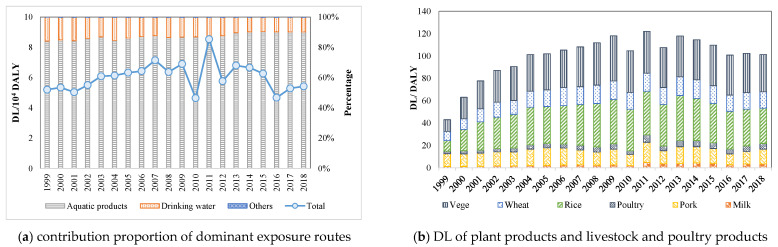
Contribution proportion of various intake exposure routes ((**a**)depicts the contribution proportion of dominant exposure routes of aquatic products, drinking water, and other exposure routes to total DL, and (**b**) depicts the contribution DL of exposures of other intake exposure routes of vegetables, wheat, rice, poultry, pork and milk).

Dietary intake of aquatic products was the dominant exposure route of EHR-IHM, accounting for 84.1–90.2% of total DL, followed by drinking water intake, accounting for 9.5–15.8%. As shown in Figure 4a, the contribution proportion of aquatic product intake increased gradually from 1999 to 2018, while those of other intake routes gradually decreased. During 1999–2018, the contribution of other intake routes, such as plant products as well as livestock and poultry products, was minimal, with a total DL of 41.5–116.5 DALY (Figure 4b). Rice was the most critical plant product for EHR-IHM. The DL of rice intake was 9.5–40.2 DALY, mainly caused by Cd emissions from industrial wastewater (accounting for 76.4%), followed by As (19.6%). The DL of intaking vegetable and wheat was 10.5–40.4 and 8.2–16.7 DALY respectively, and their primary heavy metals of the EHR-IHM were similar. Besides, the DL of intakes of pork, poultry, and milk were 9.6–18.0, 2.3–6.7, and 1.1–4.8 DALY, respectively.

Compared with aquatic products and drinking water, the main reason for the low DL of intaking rice and other agricultural products is the indirectness of the exposure processes. Heavy metals are gradually diluted in migration and transfer, and the amount of heavy metals exposed to the population is eventually small. Besides, the contribution of heavy metals to agricultural soils through irrigation was small compared to the pathways of atmospheric deposition and animal manure input. The contributions of As, Cd, Cr, Hg, and Pb through irrigation have been reported as 3.7%, 2.1%, 0.3%, 0.5%, and 0.1%, respectively [39]. However, it is worth noting that the DL caused by intake exposure of plant products as well as livestock and poultry products increased by 180.9%. Although it showed a decreasing rate of −24.6% from 2011 to 2018, the declining trend was still significantly lower than that of total DL. This phenomenon is mainly related to the accumulation of heavy metals in the soil. After years of surface water irrigation, heavy metals in industrial wastewater accumulate in the soil, are absorbed by crops, and are eventually exposed to the human body, endangering human health [40,41].

Consistent with existing research results, dietary intake is the most critical way to expose heavy metals in industrial wastewater to the human body, thus causing health damage [42,43]. People with a higher intake of aquatic products had higher exposure to heavy metals and risk levels [44]. From 1999 to 2018, the DL caused by aquatic product intake increased and then decreased, while the proportion of DL to total risk showed slow growth from 1999 to 2016. The increased intake of aquatic products and the accumulation of heavy metals were the primary reasons for this phenomenon [7]. The national per capita intake of freshwater aquatic products rose from 3.8 kg/person/year in 1999 to 7.8 kg/person/year in 2018 [45,46].

### 3.4. Identification of Distribution Characteristics of High-Risk Areas

**Figure 5:**
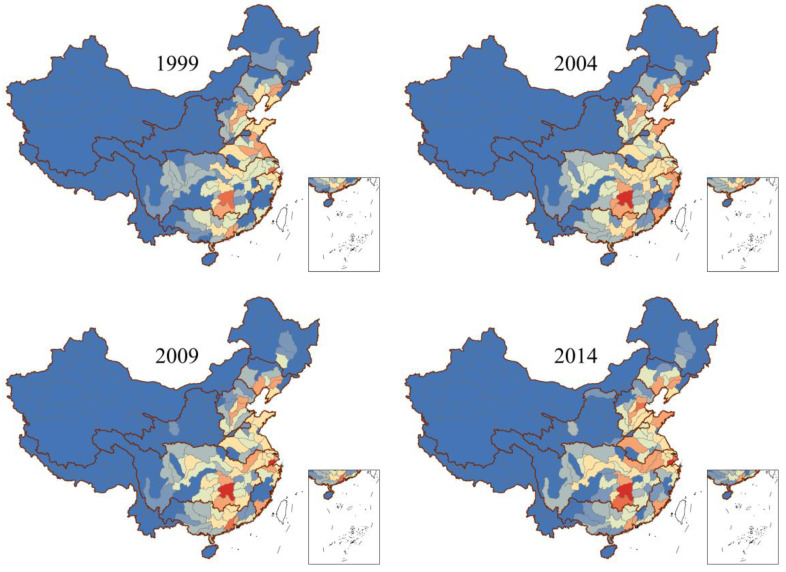

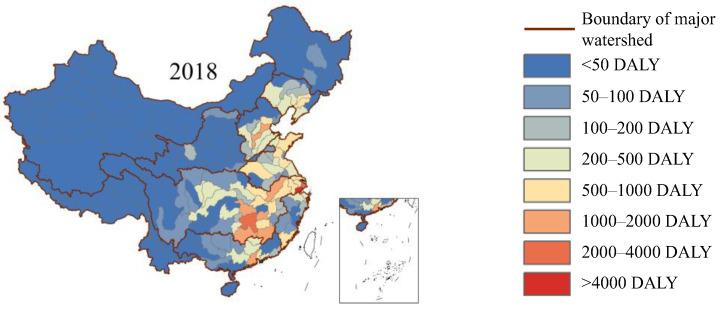
Distribution of DL caused by five heavy metal emissions in industrial wastewater.

The distribution of EHR-IHM in tertiary watersheds was calculated and visualized with ArcGIS 10.3, as displayed in Figure 5. The spatial variability of EHR-IHM in China was higher than four orders of magnitude in 1999–2018. The high-risk areas caused by heavy metal emissions in industrial wastewater were mainly concentrated in the southeast coastal regions, Bohai Rim, and the middle and lower reaches of the Yangtze River. The EHR-IHM in the Hangjiahu watershed and the downstream watershed of Xiangjiang River were highest in 1999. The EHR-IHM in the Hangjiahu watershed was mainly caused by Cr(VI) emissions from industrial wastewater. The critical heavy metal was As in the downstream watershed of Xiangjiang River. From 1999 to 2004, the EHR-IHM increased significantly, especially in the downstream watershed of Xiangjiang River, which increased from 2471.4 DALY in 1999 to 4762.1 DALY in 2004. Meanwhile, EHR-IHM in the Hangjiahu watershed decreased to 1871.3 DALY in 2004, from 2848.1 DALY in 1999. Meanwhile, the EHR-IHM in Bohai Rim and the lower reaches of the Yangtze River also obviously increased. At the same time, watersheds on the east coast decreased, such as the Lixia River watershed and the Yi-Shuhe River watershed.

From 2004 to 2014, the EHR-IHM in the middle and lower reaches of the Yangtze River and southeast coastal areas gradually increased, especially in the Northwest River Delta and Huangpu River watersheds. For example, DL in Northwest River Delta increased to 3075.7 DALY in 2009, from 1782.4 DALY in 2004, while that in Huangpu River watershed increased from 877.1 DALY in 2004 to 1924.2 DALY in 2009 and 2109.4 DALY in 2014. These increases could be attributed to the accumulation of historical heavy metal emissions and streamflow migration from upstream to downstream watersheds. Meanwhile, the DL in the Haihe River watershed, the Minnan River watershed, and the upstream watershed of Xiangjiang River were all increased in this period, mainly due to heavy metals accumulation.

It can be observed that the heavy metal pollution prevention and control in the next few years should focus on watersheds with historically high heavy metal emissions, such as the Hangjiahu watershed and Xiangjiang River watersheds. Meanwhile, southeast coastal areas located in the lower reaches of watersheds and Ganjiang River watersheds gradually increasing in EHR-IHM in recent years also need much more attention. As and Cr(VI) in industrial wastewater should be the primary-controlled heavy metals.

From the perspective of sources, the high emission intensity of industries related to raw material manufacturing, including mining and processing of non-ferrous metal ores, smelting and pressing of non-ferrous metals, and production of raw chemical materials and chemical products, was the main reason for the high DL in the downstream watershed of Xiangjiang River and Bohai Rim. These three industries together accounted for over 85% of Cd, Hg, Pb, and As emissions in the national industrial wastewater [31,47]. Moreover, the high emission intensity of metal product manufacturing, ferrous metal smelting and pressing, as well as manufacture of leather, fur, feather, and related products, was the main reason for the high DL in the lower reaches of the Yangtze River and southeast coastal regions. These three industries contributed nearly 60% to national industrial wastewater Cr(VI) emissions [47,48]. Meanwhile, stream inflow from upstream was also a critical cause for high DL in the lower reaches of the Yangtze River.

## 4. Limitations

Uncertainty of these research results is mainly due to the assumptions of the USEtox model, such as the assumption of homogenous compartments. However, despite the uncertainty, UseTox has been proven effective and reliable in assessing the toxic effects of heavy metal emissions at regional and other large scales [24]. Based on the framework of USEtox, this study evaluated the regional characteristics and inter-regional differences at the tertiary watershed scale through the construction of high-resolution spatial datasets. Meanwhile, the multimedia migration model was constructed to model the stream flows of heavy metals among watersheds. Therefore, the accuracy and reliability of simulation results are greatly improved.

However, this study only assessed the exposure and EHR-IHM caused by heavy metal emissions from industrial wastewater in 1999–2018. The emissions before 1999 were not considered. Therefore, the evaluation results may be slightly lower than the actual environmental health risk level, as the historically accumulated heavy metals before 1999 were not calculated. Besides, the implementation of heavy metal pollution control measures in some areas, such as sediment dredging, soil remediation, etc., were not considered, which could reduce the accumulation of heavy metals in environmental media. This would make the assessment results higher than the actual environmental health risk level. Due to the low accessibility of data, the evaluation results will be further improved in the future.

## 5. Conclusions

In this study, the spatio-temporal distribution of EHR-IHM during 1999–2018 in China has been revealed by coupling the accumulation process in environmental media and the cross-watershed correlations based on the framework of USEtox. Results showed that, during 1999–2018, the EHR-IHM in China increased and reached the highest DL of 8.5 × 10^4^ DALY in 2011, and then decreased. Emission of As in industrial wastewater contributed the most to the total EHR-IHM, with the proportion of 42.1–62.9%, followed by 29.7–48.4% (Cr(VI)). The contributions of Hg, Pb, and Cd emissions were small. Dietary intake of aquatic products is the dominant exposure route of EHR-IHM, accounting for 84.1–90.2% of the total, followed by drinking water intake accounting for 9.5–15.8%. The high-risk areas caused by heavy metal emissions in industrial wastewater were mainly concentrated in the southeast coastal regions, Bohai Rim, and the middle and lower reaches of the Yangtze River. Therefore, heavy metal pollution and EHR-IHM in watersheds with historically high emissions, lower reach locations, or increasing tendency should be controlled in priority.

## Figures and Tables

**Table 1:** Dominant exposure routes of agricultural foods.

Exposure Routes	Dominant Agricultural Foods
Plant products	Rice
	Wheat
	Vegetables
Livestock and poultry products	Pork
	Poultry
	Milk
Aquatic products	Aquatic products

**Table 2:** Parameters and related data sources.

Parameter	Notation	Description of Data	Source
Heavy metal emissions from industrial wastewater, kg	M_emis_	1 × 1 km	Huang et al. [31]
Irrigation volume with surface water, m^3^	V_irr,t_	Tertiary watershed	Bulletin of Water Resources
Hydraulic erosion of soil, m^3^	V_erosion_	1 × 1 km	Resource and Environment Science and Data Center
Stream flows, m^3^	V_outflow,t_	Tertiary watershed	Li et al. [32,33]
Suspended matter concentration in water, kg/m^3^	C_susp_	Tertiary watershed	China River Sediment Bulletin
Gross amount of surface water resources	V_w_	Tertiary watershed	Bulletin of Water Resources, Li et al. [32,33]
Population density	Pop	1 × 1 km	
Dietary intakes	q_inta_		Duan [34,35]

## Data Availability

The datasets generated during and/or analyzed during the current study are available from authors on reasonable request.

## Supplementary Materials

The following are available online at https://www.mdpi.com/article/10.3390/ijerph18115920/s1, Table S1: Dietary intake data of each tertiary watershed.

## References

[1] Hou D., O’Connor D., Igalavithana A.D., Alessi D.S., Luo J., Tsang D.C., Sparks D.L., Yamauchi Y., Rinklebe J., Ok Y.S. (2020). Metal contamination and bioremediation of agricultural soils for food safety and sustainability. Nat. Rev. Earth Environ..

[2] Liao J., Chen J., Ru X., Chen J., Wu H., Wei C. (2017). Heavy metals in river surface sediments affected with multiple pollution sources, South China: Distribution, enrichment and source apportionment. J. Geochem. Explor. J. Assoc. Explor. Geochem..

[3] Zhang M., Sun X., Xu J. (2020). Heavy metal pollution in the east china sea: A review. Mar. Pollut. Bull..

[4] Zhuang Q., Li G., Liu Z. (2018). Distribution, source and pollution level of heavy metals in river sediments from South China. Catena.

[5] Zhao G., Ye S., Yuan H., Ding X., Wang J., Laws E.A. (2018). Surface sediment properties and heavy metal contamination assessment in river sediments of the Pearl River Delta, China. Mar. Pollut. Bull..

[6] Xia F., Qu L., Wang T., Luo L., Chen H., Dahlgren R.A., Zhang M., Mei K., Huang H. (2018). Distribution and source analysis of heavy metal pollutants in sediments of a rapid developing urban river system. Chemosphere.

[7] Lin Y., Yu X.X., Huang L.L., Sanganyado E., Bi R., Li P., Liu W.H. (2021). Risk assessment of potentially toxic elements accumulated in fish to Indo-Pacific humpback dolphins in the South China Sea. Sci. Total Environ..

[8] Li X., Zhang J., Gong Y., Yang S., Ye M., Yu X., Ma J. (2020). Status of mercury accumulation in agricultural soils across China (1976–2016). Ecotoxicol. Environ. Saf..

[9] Liu Z., Zhang Q., Han T., Ding Y., Sun J., Wang F., Cheng Z. (2015). Heavy metal pollution in a soil-rice system in the Yangtze River Region of China. Int. J. Environ. Res. Public Health.

[10] Somma R., Ebrahimi P., Troise C., Natale G.D., Albanese S. (2021). The first application of compositional data analysis (CoDA) in a multivariate perspective for detection of pollution source in sea sediments: The Pozzuoli Bay (Italy) case study. Chemosphere.

[11] Buccino M., Daliri M., Calabrese M., Somma R. (2021). A numerical study of arsenic contamination at the Bagnoli bay seabed by a semi-anthropogenic source. Analysis of current regime. Sci. Total Environ..

[12] Manneh R., Margni M., Deschênes L. (2010). Spatial variability of intake fractions for Canadian emission scenarios: A comparison between three resolution scales. Environ. Sci. Technol..

[13] Pennington D., Margni M., Ammann C., Jolliet O. (2005). Multimedia fate and human intake modeling: Spatial versus nonspatial insights for chemical emissions in Western Europe. Environ. Sci. Technol..

[14] Zhang G., Liu H., Jia D. (2010). River basin management based on the mechanisms of water rights trading. Procedia Environ. Sci..

[15] Wu W., Wang J., Yu Y., Jiang H., Liu N., Bi J., Liu M. (2018). Optimizing critical source control of five priority-regulatory trace elements from industrial wastewater in China: Implications for health management. Environ. Pollut..

[16] Guinée J. (2001). Handbook on Life Cycle Assessment—Operational Guide to the ISO Standards.

[17] Goedkoop M., Effting S., Collignon M. (2000). The Eco-Indicator 99: A Damage Oriented Method for Life-Cycle Impact Assessment: Manual for Designers.

[18] Hauschild M., Potting J. (2005). Spatial differentiation in Life Cycle impact assessment-The EDIP2003 methodology. Environ. News.

[19] Jolliet O., Margni M., Charles R., Humbert S., Payet J., Rebitzer G., Rosenbaum R. (2003). IMPACT 2002+: A new life cycle impact assessment methodology. Int. J. Life Cycle Assess..

[20] Weidema B., Hauschild M., Jolliet O. (2007). Preparing Characterisation Methods for Endpoint Impact Assessment.

[21] Huijbregts M.A., Steinmann Z.J., Elshout P.M., Stam G., Verones F., Vieira M., van Zelm R. (2016). A Harmonized Life Cycle Impact Assessment Method at Midpoint and Endpoint Level. Report I: Characterization.

[22] Hauschild M.Z., Huijbregts M., Jolliet O., MacLeod M., Margni M., van de Meent D., Rosenbaum R.K., McKone T.E. (2008). Building a Model Based on Scientific Consensus for Life Cycle Impact Assessment of Chemicals: The Search for Harmony and Parsimony. Environ. Sci. Technol..

[23] Bare J., Young D., Qam S., Hopton M., Chief S. (2012). Tool for the Reduction and Assessment of Chemical and Other Environmental Impacts (TRACI).

[24] Pizzol M., Christensen P., Schmidt J., Thomsen M. (2011). Impacts of “metals” on human health: A comparison between nine different methodologies for Life Cycle Impact Assessment (LCIA). J. Clean. Prod..

[25] Fantke P., Bijster M., Guignard C., Hauschild M.Z., Huijbregts M., Jolliet O., Kounina A., Magaud V., Margni M., McKone T.E. (2017). USEtox® 2.0 Documentation.

[26] Ministry of Ecology and Environment of the People’s Republich of China (2011). 12th Five-Year Plan for Comprehensive Prevention and Control of Heavy Metal Pollution.

[27] Meng M., Sun R.-Y., Liu H.-W., Yu B., Yin Y.-G., Hu L.-G., Shi J.-B., Jiang G.-B. (2019). An integrated model for input and migration of mercury in chinese coastal sediments. Environ. Sci. Technol..

[28] Peng C., Wang M., Chen W. (2016). Modelling cadmium contamination in paddy soils under long-term remediation measures: Model development and stochastic simulations. Environ. Pollut..

[29] Alves R.I., Sampaio C.F., Nadal M., Schuhmacher M., Domingo J.L., Segura-Muñoz S.I. (2014). Metal concentrations in surface water and sediments from Pardo River, Brazil: Human health risks. Environ. Res..

[30] Wang Z.-H., Sun J., Wang H.-J., Liu A.-L., Zhang B., Ding G.-Q. (2019). Dietary stucture transition and development of nutrition intervention strategies in China. Acta Nutr. Sin..

[31] Huang Y., Zhou B., Li N., Li Y., Han R., Qi J., Lu X., Li S., Feng C., Liang S. (2019). Spatial-temporal analysis of selected industrial aquatic heavy metal pollution in China. J. Clean. Prod..

[32] Li Y., Zhou Q., Ren B., Luo J., Yuan J., Ding X., Bian H., Yao X. (2019). Trends and health risks of dissolved heavy metal pollution in global river and lake water from 1970 to 2017. Rev. Environ. Contam. Toxicol..

[33] Lin P., Pan M., Beck H.E., Yang Y., Yamazaki D., Frasson R., David C.H., Durand M., Pavelsky T.M., Allen G.H. (2019). Global reconstruction of naturalized river flows at 2.94 million reaches. Water Resour. Res..

[34] Duan X., Zhao X., Wang B., Zhao L., Cheng H., Cao S. (2016). Highlights of the Chinese Exposure Factors Handbook (Children).

[35] Duan X., Zhao X., Wang B., Chen Y., Cao S. (2015). Highlights of the Chinese Exposure Factors Handbook (Adults).

[36] Huang Y., Zhou B., Han R., Lu X., Li S., Li N. (2020). Spatial-temporal characteristics and driving factors of the human health impacts of five industrial aquatic toxic metals in China. Environ. Monit. Assess..

[37] Ministry of Water Resources of the People’s Republic of China (2011). 2010 China Water Resources Bulletin.

[38] Zeng X., Liu Y., You S., Zeng G., Tan X., Hu X., Hu X., Huang L., Li F. (2015). Spatial distribution, health risk assessment and statistical source identification of the trace elements in surface water from the Xiangjiang River, China. Environ. Sci. Pollut. Res..

[39] Luo L., Ma Y., Zhang S., Wei D., Zhu Y.-G. (2009). An inventory of trace element inputs to agricultural soils in China. J. Environ. Manag..

[40] Shi T., Zhang Y., Gong Y., Ma J., Wei H., Wu X., Zhao L., Hou H. (2019). Status of cadmium accumulation in agricultural soils across China (1975–2016): From temporal and spatial variations to risk assessment. Chemosphere.

[41] Shi T., Ma J., Zhang Y., Liu C., Hu Y., Gong Y., Wu X., Ju T., Hou H., Zhao L. (2019). Status of lead accumulation in agricultural soils across China (1979–2016). Environ. Int..

[42] Zhang Y., Chu C., Li T., Xu S., Liu L., Ju M. (2017). A water quality management strategy for regionally protected water through health risk assessment and spatial distribution of heavy metal pollution in 3 marine reserves. Sci. Total Environ..

[43] Agency E.E. (2018). Mercury in Europe’s Environment—A Priority for European and Global Action.

[44] Du B., Feng X., Li P., Yin R., Yu B., Sonke J.E., Guinot B., Anderson C.W.N., Maurice L. (2018). Use of mercury isotopes to quantify mercury exposure sources in inland populations, China. Environ. Sci. Technol..

[45] National Bureau of Statistics (2000). 2000 China Statistical Yearbook.

[46] National Bureau of Statistics (2019). 2018 China Statistical Yearbook.

[47] Li X., Qiao H., Wang R., Li F., Li X. (2018). Spatio-temporal data mining and modeling: Distribution pattern and governance input efficiency of heavy metal emission in industrial wastewater, China. J. Water Clim. Chang..

[48] Fan X., Luo H. (2013). Spatial and industrial distribution pattern of heavy metals emission in industrial waste water. China Environ. Sci..

